# Supplementary material for: Development and Degeneration of the Intervertebral Disc—Insights from Across Species
Source: Vet Sci. 2023 Aug 24;10(9):540. doi: 10.3390/vetsci10090540 (PMC10534844; doi:10.3390/vetsci10090540)
Supplement: Supplementary file 1 [file vetsci-10-00540-s001.zip › vetsci-2516046-supplementary.pdf]

Supplementary table1: Abbreviations of genes or proteins

**Abbreviation as Referenced**	**Official Abbreviation**	**Official Full Name**	**NCBI Gene ID**
Acan	Acan	aggrecan	Rat: 58968
Acp5	Acp5	acid phosphatase 5	Rat: 25732
ADAMTS-4	ADAMTS4	ADAM metallopeptidase with thrombospondin type 1 motif 4	Human: 9507<br>Bovine: 286806
ADAMTS5	ADAMTS5	ADAM metallopeptidase with thrombospondin type 1 motif 5	Human: 11096<br>Mouse: 23794
Adamts17	ADAMTS17	ADAM metallopeptidase with thrombospondin type 1 motif 17	Bovine: 538131
ADIRF	ADIRF	adipogenesis regulatory factor	Human: 10974
Akap12	Akap12	A-kinase anchoring protein 12	Rat: 83425
Aldh1a1	Aldha1a1	aldehyde dehydrogenase 1 family, member A1	Rat: 24188
Ang1	ANGPT1	angiopoietin 1	Human: 284
Anxa3	Anxa3	annexin A3	Mouse: 11745
Atp6v1g3	ATP6V1G3	ATPase H+ transporting V1 subunit G3	Bovine: 613925
Basp1	BASP1	brain abundant, membrane attached signal protein 1	Human: 10409<br>Rat: 70350
BDNF	BDNF	brain derived neurotrophic factor	Porcine: 617701
Bmp7	Bmp7	bone morphogenetic protein 7	Rat: 85272
Bpifa1a	Bpifa1	BPI fold containing family A member 1	Human: 51297
Bpifb1	Bpifb1	BPI fold containing family B, member 1	Rat: 499926
C10ORF10	DEPP1	DEPP autophagy regulator 1	Human: 11067
C1qtnf3	C1QTNF3	C1q and TNF related 3	Bovine: 531659
C2ORF40	ECRG4	ECRG4 augurin precursor	Human: 84417
Ca3	Car3	carbonic anhydrase 3	Mouse: 12350
CALCR	CALCR	calcitonin receptor	Goat: 102184585
CAPS	CAPS	calcyphosine	Human: 828
CASPASE-1	CASP1	caspase 1	Human: 834
Caspase-12	Casp12	caspase 12	Rat: 156117
Caspase-3	CASP3	caspase 3	Bovine: 408016

CAV1	CAV1	caveolin 1	Goat: 100860749
CCL20	CCL20	C-C motif chemokine ligand 20	Human: 6364
CCL5	CCL5	C-C motif chemokine ligand 5	Human: 6352
CCL7	CCL7	C-C motif chemokine ligand 7	Human: 6354
Ccn3	CCN3	cellular communication network factor 3	Bovine: 505727
CCR7	CCR7	C-C motif chemokine receptor 7	Human: 1236
CD109	CD109	CD109 antigen	Human: 135228<br>Mouse: 235505
CD155	PVR	PVR cell adhesion molecule	Human: 5817
CD163	CD163	CD163 molecule	Human: 9332
CD206	MRC1	mannose receptor C-type 1	Human: 4360
CD221	IGF1R	insulin like growth factor 1 receptor	Human: 3480
CD24	CD24	CD24 molecule	Human: 100133941<br>Rat: 25145
CD44	Cd44	CD44 antigen	Mouse: 12505
CD55	CD55	CD55 molecule	Bovine: 518609
CD81	CD81	CD81 antigen	Mouse: 12520
CD90	THY1	Thy-1 cell surface antigen	Human: 7070<br>Mouse: 21838<br>Bovine: 614712
Cdh2	Cdh2	cadherin 2	Human: 1000<br>Mouse: 12558<br>Bovine: 281062
Chcd7	Chcd7	coiled-coil-helix-coiled-coil-helix domain containing 7	Human: 79145
CHI3L1	CHI3L1	chitinase 3 like 1	Human: 1116
CHI3L2	CHI3L2	chitinase 3 like 2	Human: 1117
Cilp	Cilp	cartilage intermediate layer protein	Rat: 315761
CLC	CLC	Charcot-Leyden crystal galectin	Monkey: 102120083
CLEC3A	CLEC3A	C-type lectin domain family 3 member A	Human: 10143
CLND1	CLDN1	Claudin 1	Human: 9076
CNTFR	Cntfr	ciliary neurotrophic factor receptor	Mouse: 12804
Col10a1, Col10	Col10a1	collagen, type X, alpha 1	Mouse: 12813<br>Rat: 25681

Col11a1	Col11a1	collagen type XI alpha 1 chain	Rat: 25654
Col12a1	Col12a1	collagen, type XII, alpha 1	Human: 1303<br>Mouse: 12816
COL2A1, Col2	COL2A1	Collagen type II alpha chain 1	Human: 1280<br>Mouse: 12824<br>Rat: 25412
COL5A1	COL5A1	collagen type V alpha 1 chain	Human: 1289<br>Mouse: 12831
Col5a2	Col5a2	collagen, type V, alpha 2	Mouse: 12832
Col8a1	Col8a1	collagen type VIII alpha 1 chain	Rat: 304021
Comp	COMP	cartilage oligomeric matrix protein	Human: 1311<br>Bovine: 281088
Cox2	COX2	cytochrome c oxidase subunit II	Dog: 804479<br>Bovine: 3283880
COX2	MT-CO2	mitochondrially encoded cytochrome c oxidase II	Human: 4513
Cp	CP	ceruloplasmin	Bovine: 514194
Creld2	CRELD2	cysteine rich with EGF like domains 2	Bovine: 515222
CRP	CRP	C-reactive protein	Human: 1401
CTGF	CCN2	cellular communication network factor 2	Human: 374135
CTSK	CTSK	cathepsin K	Human: 1513<br>Bovine: 513038
CXCL12	CXCL12	C-X-C motif chemokine ligand 12	Human: 6387
Cxcl3	Cxcl3	C-X-C motif chemokine ligand 3	Rat: 171551
CXCL6	CXCL6	C-X-C motif chemokine ligand 6	Human: 6372
Cytochrome c	Cycs	Cytochrome c, somatic	Rat: 25309
Dcn	DCN	decorin	Human: 1634<br>Bovine: 280760
Dnajc3	DNAJC3	DnaJ heat shock protein family (Hsp40) member C3	Bovine: 286770
ENG	ENG, CD105	endoglin	Human:2022<br>Mouse: 13805<br>Goat: 102169304
EPYC	EPYC	epiphycan	Human: 1833
ERK1	MAPK3	mitogen-activated protein kinase 3	Human: 5595
ERK2	MAPK1	mitogen-activated protein kinase 1	Human: 5594

Fas	Fas	Fas cell surface death receptor	Human: 355
FBLN1	FBLN1	fibulin 1	Human: 2192
FBXO2	FBXO2	F-box protein 2	Human: 26232
FGFBP2	FGFBP2	fibroblast growth factor binding protein 2	Human: 83888
Fibin	Fibin	fin bud initiation factor homolog	Rat: 499856
Fn1	FN1	fibronectin 1	Human: 2335<br>Mouse: 14268<br>Rat: 25661<br>Bovine: 280794
FRZB	FRZB	frizzled related protein	Human: 2487
FTH	Fth1	ferritin heavy chain 1	Rat: 25319
GADD153	Ddit3	DNA-damage inducible transcript 3	Rat: 29467
Gasdermin-D	Gsdmd	Gasdermin D	Human: 79792<br>Mouse: 69146<br>Rat: 315084
GCH1	GCH1	GTP cyclohydrolase 1	Human: 2643
Gfap	Gfap	glial fibrillary acidic protein	Equine: 100033970
Gli1	GLI1	GLI family zinc finger 1	Human: 2735<br>Mouse: 14632<br>Bovine: 517588<br>Monkey: 102136450
Gli3	GLI3	GLI family zinc finger 3	Human:<br>Mouse: 14634<br>Bovine: 785371
Glutamic-oxalocetictransaminase	GOT1	glutamic-oxaloacetic transaminase 1	Equine: 100060793
GPX4	Gpx4	glutathione peroxidase 4	Rat: 29328
GRB10	GRB10	growth factor receptor bound protein 10	Human: 2887
GRP78	Hspa5	heat shock protein family A (Hsp70) member 5	Rat: 25617
Gsn	GSN	gelsolin	Bovine: 535077
H2ac18	H2AC18	H2A clustered histone 18	Bovine: 541108
HIF1α	HIF1A	hypoxia inducible factor 1 subunit alpha	Monkey: 101866092
HMGA1	HMGA1	high mobility group AT-hook 1	Human: 3159
hydroxylysine	HYKK	hydroxylysine kinase	Equine: 100060343
ID1	ID1	inhibitor of DNA binding 1	Human: 3397
ID3	ID3	inhibitor of DNA binding 3	Human: 3399

IFNy	IFNG	interferon gamma	Human: 3458
IGF1	IGF1	insulin like growth factor 1	Human: 3479
Igfbp5	Igfbp5	insulin-like growth factor binding protein 5	Rat: 25285
Igfbp6	IGFBP6	insulin like growth factor binding protein 6	Bovine: 404186
IHH	IHH	Indian hedgehog signaling molecule	Monkey: 102136990
IL10	IL10	interleukin 10	Human: 3586<br>Rat: 25325
Il11	Il11	Interleukin 11	Rat: 171040
IL12	Il12b	Interleukin 12B	Rat: 64546
IL17	IL17a	Interleukin 17a	Human: 3605
IL18	IL18	interleukin 18	Human: 3606<br>Rat: 29197
IL1β	IL1B	interleukin 1 beta	Human: 3553<br>Porcine: 281251<br>Bovine: 281251<br>Rat: 24494
IL4	IL4	Interleukin 4	Human: 3565
IL6	IL6	interleukin 6	Human: 3569<br>Porcine: 280826<br>Bovine: 280826
IL8	CXCL8	C-X-C motif chemokine ligand 8	Human: 3576<br>Dog: 403850<br>Bovine: 280828
Inhba	Inhba	inhibin subunit beta A	Rat: 29200
ISL1	ISL1	ISL LIM homeobox 1	Human: 3670
KRT18	Krt18	keratin 18	Human: 3875<br>Mouse:16668<br>Equine: 100063542
Krt19	Krt19	keratin 19	Human: 3880<br>Mouse: 16669
Krt7	Krt7	keratin 7	Rat: 300242
Krt8	Krt8	keratin 8	Human: 3856<br>Mouse: 16691<br>Bovine: 281269
Lam1	LAM1	Lam1p	S. cerevisiae: 856560
LCN2	LCN2	lipocalin 2	Human: 3934
Lect1	Cnmd	chondromodulin	Mouse: 16840
LGALS1	LGALS1	galectin 1	Human: 3956<br>Bovine: 326598
LIF	LIF	LIF interleukin 6 family cytokine; Leukemia Inhibitory Factor	Human: 3976<br>Mouse: 16878

Loc101904175	LOC101904175	uncharacterized LOC101904175	Bovine: 101904175
Lum	Lum	lumican	Mouse: 17022<br>Rat: 81682
LYN	LYN	LYN proto-oncogene, Src family tyrosine kinase	Human: 4067
MAP1B	MAP1B	microtubule associated protein 1B	Human: 4131
MFG-E8	MFGE8	milk fat globule EGF and factor V/VIII domain containing	Human: 4240
MGP	MGP	matrix Gla protein	Human: 4256<br>Bovine: 282660
MGST1	MGST1	microsomal glutathione S-transferase 1	Human: 4257
MMP1	MMP1	matrix metallopeptidase 1 (interstitial collagenase)	Bovine: 281308
MMP13	MMP13	matrix metallopeptidase 13	Human: 4322
Mmp3	MMP3	matrix metallopeptidase 3	Bovine: 4314<br>Rat: 171045
MMP7	MMP7	matrix metallopeptidase 7	Human: 4316
MMP8	MMP8	matrix metallopeptidase 8	Human: 4317
MMP9	MMP9	matrix metallopeptidase 9	Human: 4318
Mmp13	Mmp13	matrix metallopeptidase 13	Rat: 171052<br>Human: 4322
MSMP	MSMP	microseminoprotein, prostate associated	Human: 692094
MT-ND2	MTND2	mitochondrially encoded NADH dehydrogenase 2	Human: 4536
MT1F	MT1F	metallothionein 1F	Human: 4494
MT1G	MT1G	metallothionein 1G	Human: 4495
MT2A	MT2A	metallothionein 2A	Human: 4502
MTCYB	MT-CYB	mitochondrially encoded cytochrome b	Human: 4519
MyD88	MYD88	MYD88 innate immune signal transduction adaptor	Human: 4615
Myoc	Myoc	myocilin	Rat: 81523
NAMPT	NAMPT	nicotinamide phosphoribosyltransferase	Human: 10135
Ncdn	Ncdn	neurochondrin	Rat: 89791<br>Human: 23154
NF-kB	NFKB1	nuclear factor kappa B subunit 1	Human: 4790<br>Mouse: 18033<br>Rat: 81736
NGF	NGF	nerve growth factor	Human: 4803

			Bovine: 281350<br>Porcine: 281350
Ngfr	NGFR	nerve growth factor receptor	Bovine: 353110
NLRP3	NLRP3	NLR family pyrin domain containing 3	Human: 114548<br>Rat: 287362
NOD2	NOD2	nucleotide binding oligomerization domain containing 2	Human: 64127
Noto	NOTO	notochord homeobox	Human: 344022<br>Mouse: 384452<br>Bovine: 100335771
NRP-1	NRP1	neuropilin 1	Human: 8829<br>Rat: 246331
p38 MAPK	Mapk14	mitogen activated protein kinase 14	Human: 1432<br>Mouse: 26416<br>Rat: 81649
p53	TP53	tumor protein p53	Human: 7157<br>Mouse: 22059<br>Bovine: 281542
Pdia4	PDIA4	protein disulfide isomerase family A member 4	Bovine: 415110
PECAM1	PECAM1	platelet and endothelial cell adhesion molecule 1	Goat: 102179808
PI3K	PIK3CB	phosphatidylinositol-4,5-bisphosphate 3-kinase catalytic subunit beta	Human: 5291
PLA2G2A	PLA2G2A	phospholipase A2 group IIA	Human: 5320
pNF-H	NEFH<br>*p indicates phosphorylation	neurofilament heavy chain	Human: 4747
PRELP	PRELP	proline and arginine rich end leucine rich repeat protein	Human: 5549
PRG4	PRG4	proteoglycan 4	Human: 10216<br>Mouse: 96875<br>Bovine: 280867
PROCR	PROCR	protein C receptor	Goat: 102171169
PRPH	PRPH	Peripherin	Human: 5630
Prrg4	Prrg4	proline rich and Gla domain 4	Rat: 499847
PTGS2	Ptgs2	prostaglandin-endoperoxide synthase 2	Rat: 29527

Ptprc	Ptprc	protein tyrosine phosphatase receptor type C	Human: 19264
Rab38	Rab38	RAB38, member RAS oncogene family	Rat: 252916
Rcn2	RCN2	reticulocalbin 2	Bovine: 512717
Ripk4	Ripk4	receptor-interacting serine-threonine	Rat: 304053
RTN1	RTN1	Reticulon 1	Human: 6252
S100A1	S100A1	S100 calcium binding protein A1	Human: 6271
S100A2	S100A2	S100 calcium binding protein A2	Human: 6273
S100b	S100B	S100 calcium binding protein B	Bovine: 525716
Scx	SCX	scleraxis bHLH transcription factor	Bovine: 615157
SEMA3A	SEMA3A	semaphorin 3A	Human: 10371
SEMA3C	SEMA3C	semaphorin 3C	Goat: 102183759
SHH	SHH	Sonic hedgehog signaling molecule	Human: 6469<br>Mouse: 20423<br>Bovine: 286821<br>Monkey: 102120970
SLC7A2	SLC7A2	solute carrier family 7 member 2	Human: 6542
Slca1	Slc2a1	solute carrier family 2 member 1	Human: 6513
SLPI	SLPI	secretory leukocyte peptidase inhibitor	Human: 6590
SMO	SMO	smoothened, frizzled class receptor	Human: 6608<br>Mouse: 319757<br>Monkey: 102121742
Snap25	SNAP25	synaptosome associated protein 25	Human: 6616<br>Bovine: 540853
Snorc	SNORC	secondary ossification center associated regulator of chondrocyte maturation	Bovine: 618319
Sod2	SOD2	superoxide dismutase 2	Human: 6648<br>Bovine: 281496
Sostdc1	SOSTDC1	sclerostin domain containing 1	Bovine: 523184
SOX10	SOX10	SRY-box transcription factor 10	Human: 6663
Sox2	SOX2	SRY-box transcription factor 2	Human: 6657<br>Bovine: 784383

Sox9	Sox9	SRY-box transcription factor 9	Human: 6662<br>Mouse: 20682<br>Bovine: 100336535<br>Rat: 140586
SPARC	SPARC	secreted protein acidic and cysteine rich	Human: 6678
Spp1	SPP1	secreted phosphoprotein 1	Bovine: 281499<br>Human: 6696
SPTSSB	SPTSSB	serine palmitoyltransferase small subunit B	Human: 165679
SSP1	SENP6	SUMO specific peptidase 6	Human: 26054
STMN2	STMN2	stathmin 2	Human: 11075
SYF2	SYF2	SYF2 pre-mRNA splicing factor	Human: 25949
TAF1D	TAF1D	TATA-box binding protein associated factor, RNA polymerase I subunit D	Human: 79101
TBXT	TBXT, T	T-box transcription factor T	Human: 6862<br>Mouse: 20997<br>Rat: 360737
TGFβ	TGFB1	transforming growth factor beta 1	Human: 7040<br>Mouse: 21803<br>Bovine: 282089
THBS2	THBS2	thrombospondin 2	Human: 7058
Thbs4	Thbs4	thrombospondin 4	Human: 7060<br>Rat: 29220
Tie1	TIE1	tyrosine kinase with immunoglobulin like and EGF like domains 1	Bovine: 280941
Tie2 or TEK	TEK	TEK receptor tyrosine kinase	Human: 7010
Timp1	Timp1	TIMP metallopeptidase inhibitor 1	Rat: 7076
Timp3	TIMP3	TIMP metallopeptidase inhibitor	Bovine: 282094
TLR4	TLR4	toll like receptor 4	Human: 7099
TMED	TMED1	transmembrane p24 trafficking protein 1	Human: 11018A
Tmnd	Tmnd	tenomodulin	Mouse: 64103
TNFα	TNF	tumor necrosis factor	Human: 7124<br>Mouse: 21926<br>Bovine: 280943<br>Goat: 100861232<br>Dog: 403922

TNFR1	TNFRSF1A	TNF receptor superfamily member 1A	Human: 7132
TNFRSF1B	TNFRSF1B	TNF receptor superfamily member 1B	Human: 7133
TNFSF10	TNFSF10	TNF superfamily member 10	Goat: 102173335
Tnmd	Tnmd	tenomodulin	Bovine: 781292<br>Rat: 64104
TNC		Tenascin-C, cytoactin	Human: 3371<br>Mouse: 21923<br>Bovine: 540664
UPP1	UPP1	uridine phosphorylase 1	Human: 7378
UTS2R	Utsr2	urotensin 2 receptor	Human: 2837<br>Mouse: 217369<br>Bovine: 286969
Vdr	Vdr	vitamin D receptor	Rat: 22337
VEGF	VEGFA	vascular endothelial growth factor A	Human: 7422<br>Goat: 100860957
VIM	VIM	vimentin	Human: 7431
Wdr73	WDR73	WD repeat domain 73	Bovine: 783802
Wnt4	WNT4	Wnt family member 4	Rat: 84426
Zscan10	Znf206	zinc finger and SCAN domain containing 10	Mouse: 332221<br>Bovine: 538853

Supplementary Table: Table information compiled from NCBI Gene [1] represents the agreed upon abbreviation and name of each gene in addition to the gene ID.

1. Gene [Internet]. Bethesda (MD): National Library of Medicine (US), National Center for Biotechnology Information; 2004 – cited 2023 Jun. 30. Available from: https://www.ncbi.nlm.nih.gov/gene/
